# Anticipated adaptation or scale recalibration?

**DOI:** 10.1186/1477-7525-11-171

**Published:** 2013-10-18

**Authors:** Yvette Edelaar-Peeters, Anne M Stiggelbout

**Affiliations:** 1 Department of Medical Decision Making, Leiden University Medical Centre, P.O. Box 9600, 2300, RC, Leiden, The Netherlands

**Keywords:** Scale recalibration, Adaptation, Time tradeoff, Visual analogue scale, Response shift and spinal cord injury, Health state utility

## Abstract

**Background:**

The aim of our study was to investigate anticipated adaptation among patients in the subacute phase of Spinal Cord Injury (SCI).

**Methods:**

We used an observational longitudinal design. Patients with SCI (N = 44) rated their actual, previous and expected future Quality of Life (QoL) at three time points: within two weeks of admission to the rehabilitation center (RC), a few weeks before discharge from the RC, and at least three months after discharge. We compared the expected future rating at the second time point with the actual ratings at the third time point, using student’s t-tests. To gain insight into scale recalibration we also compared actual and previous ratings.

**Results:**

At the group level, patients overpredicted their improvement on the VAS. Actual health at T3(M = 0.65, sd =0.20)) was significantly lower than the predicted health at T1 of T3 (M = 0.76, sd = 0.1; t(43) = 3.24, p < 0.01), and at T2 of T3(M = 0.75,sd = 0.13; t(43) = 3.44, p < 0.001). Similarly the recalled health at T3 of T2 (M = 0.59, sd = 0.18) was significantly lower than the actual health at T2 (M = 0.67, sd = 0.15; t(43) = 3.26, p <0.01). Patients rated their future and past health inaccurately compared to their actual ratings on the VAS. In contrast, on the TTO patients gave accurate estimates of their future and previous health, and they also accurately valued their previous health. Looking at individual ratings, the number of respondents with accurate estimates of their future and previous health were similar between the VAS and TTO. However, the Bland-Altman plots show that the deviation of the accuracy is larger for the TTO then the VAS. That is the accuracy of 95% of the respondents was lower in the TTO then in the VAS.

**Conclusions:**

Patients at the onset of a disability were able to anticipate adaptation. Valuations given on the VAS seem to be biased by scale recalibration.

## Introduction

Patients experiencing an illness or disability generally give high Quality of Life (QoL) ratings compared to ratings given by people imagining having such an illness or disability [1]. Such an underestimate of QoL by people imagining illness or disability is common. Previous research suggests that this is due to people focusing on negative aspects of a disability (focusing illusion) and a failure to anticipate adaptation [2,3]. Patients adapt physically as well as psychologically to their disability, leading to an improvement of their QoL. People imagining a disability have a tendency to focus on the incident and fail to anticipate this ability to adapt. As a consequence, they misjudge their long-term emotional responses to such events [4].

Besides underprediction of QoL, overprediction has also been shown. Peeters et al. [5] showed that patients expected greater improvement of their QoL at the onset of a disability than they actually got. These findings may have been led by a measurement bias called scale recalibration. Although patients in this study were recovering from surgery they did not report any actual improvement over time. If the findings of this study were led by scale recalibration, it will become impossible to draw conclusions about anticipated adaptation. We cannot differentiate between scale recalibration and anticipated adaptation.

Together with reprioritization and reconceptualization, scale recalibration is one of the three constituents of response shift. Response shift was initially described by Golembiewski et al. [5]. Sprangers and Schwartz introduced it into QoL research [6]. The first construct, reprioritization describes the process in which a respondent changes the importance of the domains that constitute the underlying construct. For instance, a respondent might first claim that her QoL is mainly based on her physical wellbeing whereas overtime she might find that her physical wellbeing is not that important any more for her QoL. The second contruct, reconceptualization, is a redefinition of the construct QoL. That is, a respondent might initially assume that QoL is based on physical health but overtime she might change her definition of QoL in which physical health is not part of the construct anymore [6]. The third construct, scale recalibration is a process in which the intervals of a scale used to measure a dimension are recalibrated after an intervening event. In the field of QoL research, this event may be a course of treatment, the development of a disability, or the diagnosis of an illness. Such event might cause the intervals on a scale to expand or contract, depending on the change in context [5]. Likert scales have proven to be sensitive to scale recalibration [7].

Constituents of response shift are strongly related to adaptation. Response shift has been described an effect of adaptation [8]. The process of adaptation to chronic illness and disability is dynamic, nonlinear and unpredictable, with individuals undergoing several transitions, with the last transition evidenced by a reconstructed self-identity including appreciation of diverse activities and life goals [9]. Quotes reported in qualitative studies of adaptation (“changing my habits, which means that I changed, I no longer do the same work, I used to work a lot before” [10]), are similar to quotes reported in a qualitative studies on response shift (“I can do some work in the house, like sweeping the floor, so I’m not limited” [11]). This has been argued to be mostly true for the concepts reconceptualization and reprioritization, as scale recalibration refers to the measurement scale [12]. This distinction between scale recalibration on the one hand and reconceptualization and reprioritization on the other, in relation to adaptation has been criticized, with some arguing that the changes in the scale are also part of the adaptation process. As can be read from a thorough discussion in the literature there are different theoretical points of view on the role for scale recalibration [12-16]. As response shift literature argues that scale recalibration is part of the response shift and therefore part of the effect of adaptation [6,8], others have argued that scale recalibration should be regard as measurement error [7,12].

In the context of this study, we regard scale recalibration as measurement bias and reconceptualization and reprioritization as an effect of adaptation. We do not make a distinction between physical and psychological adaptation, therefore the concept 'adaptation’ refers to aspects such as learning how to handle a wheelchair, finding new hobbies, and learning how to live with a new situation.One type of measures used in the field of QoL research, are preference measurements, or health state valuations, mostly used in the field of decision-making. Whereas descriptive QoL measurements only describe QoL, preference measurements measure both QoL and the valuation of QoL, relative to perfect health and death [17]. One of these preference measurements is the Visual Analog Scale (VAS). In the VAS, patients give valuations of their health by placing a mark on a 100 mm. horizontal or vertical line ranging from perfect health to death. Just like the Likert-scale method the VAS is a rating scale, but mostly without labeling intervals; (as an exception the VAS of the EQ-5D used to estimate health state tariffs does have labeling intervals [18]). When labeling intervals are missing, respondents have the tendency to divide the scale using their own 'intervals’ [19]. Probably these personal 'intervals’ can expand or contract just like the intervals of a likert scale [5]. Lacey et al. [7] found support for the effect of scale recalibration on VAS ratings. Its feasibility makes the VAS a popular method, but a limitation of the VAS is that it does not provide a trade-off valuation, which is required in e.g. the assessment of quality-adjusted life years for decision models or cost-effectiveness analysis [20]. Another instrument often used in health state valuations which does suit this goal is the Time Trade-Off (TTO) [21]. In the TTO, participants choose between a number of life years in their current state of health and a shorter lifespan in perfect health. The severity of the state of health estimated is based on the number of years a person is willing to trade for perfect health. TTO ratings are measured on a time scale, usually in life years. On a time scale, the intervals are stable. It is hard to imagine how 'time’ as a response scale can be the subject of recalibration, unless the survival time prognosis of the respondent changes dramatically. Theoretically, the TTO therefore seems less vulnerable to scale recalibration than the VAS, yet no empirical evidence has been described to ground this assumption.

The aim of our study is to attempt to disentangle scale recalibration and inability to anticipate one’s adaptation, in patients with SCI. We asked patients to predict future and recall former state of health including physical, psychological and social aspects using a VAS and a TTO. We will use a method similar to the then-test assessing improvement in QoL [22] to examine scale recalibration and anticipated adaptation. The then test gives information at the group level which can be used to guide treatment decisions at the group level but will have measurement error when decisions are made at the individual level [23]. Therefore, individual methods such as the Bland-Altman plot will be used as well. Since we expect valuations given on a TTO to be less sensitive to scale recalibration than valuations given on a VAS, as explained above, we compared results for these two measures. Previous research among patients with SCI revealed that for some patients the change over time in satisfaction with life -measured with a likert-scale- was due to scale recalibration [24]. Others found that patients with SCI changed their priorities over time [25]. In the current study, we expected patients to overpredict changes in their state of health over time on both the VAS and TTO, if they overestimated their ability to adapt. However, if they correctly estimate their ability to adapt they will make correct predictions of changes in their state of health on the TTO. On the VAS they will still make overpredictions of these changes, due to scale recalibration.

## Methods

### Participants and procedures

Patients between 18 and 75 years old with SCI who were able to understand and speak Dutch, with sub-acute SCI causing functional losses and problems with daily activities, were asked to participate in our study. Patients with minor functional losses (who had neither problems with walking ability nor problems with bladder or bowel functions) as well as patients with severe emotional or cognitive problems were excluded. Eligible patients were contacted by their attending physician or psychologist in the first few weeks of admission. We collaborated with six Rehabilitation Centers (RCs) in The Netherlands specializing in SCI. Patients who consented to be interviewed were contacted by one of the interviewers.

Patients were interviewed at three time points. These time points were based on clinical moments given by the rehabilitation schedule. This meant that the interviews were scheduled around major events in the rehabilitation trajectory similar for all patients. The first interview took place at the RC as soon as possible after admission, the second interview during active rehabilitation, also at the RC, and the third after discharge when patients were accustomed to their home situation. This interview took place at home or during an out-patient clinic visit to the RC. Along with these clinical moments, a time frame was used; the first interview took place within the first four weeks of admission, the second interview at least two weeks before discharge and a month after the first interview, and the third interview took place at least six months after discharge. Since the rehabilitation period of patients with tetraplegia is generally longer, the time between interviews differed for patients with paraplegia and patients with tetraplegia. For patients with paraplegia, the schedule was therefore an interval of three months between the first and second interview and an interval of one year between the first and third interview. For patients with tetraplegia, the interview schedule included an interval of six months between the first and second interview and an interval of 18 months between the first and third interview. A great deal of effort was put into interviewing all patients within these time frames; exceptions that had to be made are described in the results section. The medical ethics committee of the Leiden University Medical Center (LUMC) and the local ethics committees of the RCs approved the study protocol.

### The interview

At all three time points, a face-to-face interview was conducted by one of two trained interviewers following a strict interview protocol. Overall the same questions were asked at the three time points, but small changes were made when necessary due to the different situation patients were in. In the introduction of the interview, patients were instructed not only to take their illness into account in answering questions about their health, but to incorporate the limitations caused by their injury as well. Patients gave a valuation of their own health of the previous week using a VAS and a TTO. In the first and second interviews this was followed by a (predicted) valuation of their state of health at least six months after discharge from the RC when the patient was expected to be home; approximately the time of the third interview. In the third interview, the valuation of their own state of health at that moment was followed by a valuation of their own health as they recalled it to be during the second interview.

### Assessments

#### *The time trade-off (TTO)*

Patients rated how many years (x) of their remaining life expectancy (y) they were willing to trade in order to obtain perfect health. Utility was calculated as y-x/y. Life expectancy was derived from Dutch life expectancy tables for their gender and age category [26]. The interviewer asks a participant to choose between a number of years, i.e. their remaining life expectancy, living in the health state to be valued or living a shorter period of time in perfect health. The time in perfect health is varied to obtain an indifference point, the number of years in perfect health equal to a higher number of years in the health state to be valued. For example if a respondent has a life expectancy of 20 years the interviewer asks the respondent to choose between 20 years living in the health state to be valued or living for instance 10 years in perfect health. If the respondent chooses for the health state to be valued the duration in perfect health will increase if the respondent chooses for perfect health the duration will decrease. By changing the duration of perfect health an indifference point can be sought. The indifference point was sought through the bisection method. TTO utilities were elicited using a time-line and board. The descriptions of the health states are placed on the board, and with the sliding time-line the duration of the health state was made visible. The health states were perfect health and the patients’ own health of the previous week, their future, or their previous state of health, respectively. Perfect health was described as full well-being in physical, psychological, and social functioning. Given the severity of SCI and the emotional status of the patients, the lowest trade-off was set at three months living in perfect health.

#### *The visual analogue scale (VAS)*

The VAS is a 100 mm horizontal line anchored by death and perfect health. Perfect health was again described as full well-being in physical, psychological and social functioning. The valuation of their own health of the previous week (actual health), of their expected future state of health, and previous state of health were elicited by placing a mark between death and perfect health, and calculated as #mm/100.

### Data analyses

Prior to the main analyses, all variables were examined for normality by relating the level of skewness, and kurtosis to the standard deviation. All variables were normally distributed. We used Students’ t-tests to assess differences between the original scores, which we will call the 'actual change’ in health state valuations and the difference between the original scores and scores for predicted health state which we will call the “accuracy in predicting” and the difference between the original scores and the scores for the recalled health which we will call “accuracy in recalling”.

Individual accuracy was tentatively (because of the known moderate ICC reliability of the methods [27,28]) examined by examining the percentage of respondents who made correct estimates. Given that valuations given on the VAS and TTO both can be calculated to more than two decimals specific, we allowed for an individual rating-error of 0.100. That is if the difference between two ratings was within 0.100 we report this as an equal rating. To further examine the individual accuracy Bland-Altman plots were prepared which visualize the accuracy by showing the 95% confidence interval.

## Results

Physicians or psychologists approached 74 patients who met our inclusion criteria. Of these, 14 patients refused due to personal reasons. Ten of these patients refused because they did not want to participate, two patients refused because of their religion, and two patients found it difficult to talk about their state of health. No differences in age and gender were found between patients refusing to participate and patients who participated in the first interview. We had no information on the medical condition of patients who refused.

In total 60 (74-14) patients (81%) agreed to participate and were interviewed at T1. At T2 four patients withdrew for personal reasons and one patient could not be contacted. Three patients had to be excluded:one patient was excluded due to an infection that made active rehabilitation impossible and two patients were not interviewed because the time between the first interview and discharge had been less than a month. Two patients had incomplete data because at the time of their interview the prediction of the future state of health had not been included as yet. At T3, four patients dropped out (two withdrew due to major pain, one had passed away, and one could not be interviewed due to logistic reasons). Finally two respondents were excluded from the analysis who did not want to make a prediction of their future health. In total 44 patients of the initially 74 approached (59%) were included in our analyses, 73% of the 60 patients who took part in the first interview. No statistically significant differences in gender, marital status, children, education level, age and cause of injury were found between patients excluded in the second or third interview and patients included. However, a difference was found in type of injury: more patients with incomplete tetraplegia had dropped out.

Tables 1 and 2 show the demographic characteristics and mean valuations of the included patients.

**Table 1:** Demographic characteristics of patients with spinal cord injury participating in this study

**Categorical variables**	**N**	**%**
Gender		
Female	15	34
Male	29	66
Marital status		
Married	26	59
Divorced/Widow	9	21
Single	9	21
Children		
Yes	25	57
No	19	43
Education		
Nine years or less	11	25
Between 10 - 12 years	18	41
13 years or more	15	34
Type injury		
Incomplete paraplegia	13	30
Complete paraplegia	19	43
Incomplete tetraplegia	10	23
Complete tetraplegia	2	5
Cause of injury		
Accident	26	59
Illness	10	23
Surgery	8	18
Help answering VAS^†^		
No help	32	73
Help needed	11	25

^†^Some participants with problems in their upper limbs were assisted when answering the Visual Analogue Scale (VAS).

**Table 2:** Continuous characteristics of the patients with spinal cord injury participating in this study

**Continuous variables**	**Mean**	**SD**
Age	46	15
Weeks between incident and first interview		
Paraplegia	11	5
Tetraplegia	11	4
Weeks between first and second interview		
Paraplegia	12	5
Tetraplegia	25	6
Weeks between discharge and third interview		
Paraplegia	28	20
Tetraplegia	36	24

### Accuracy of predicted and recalled health state valuations compared to actual ratings

**Figure 1:**
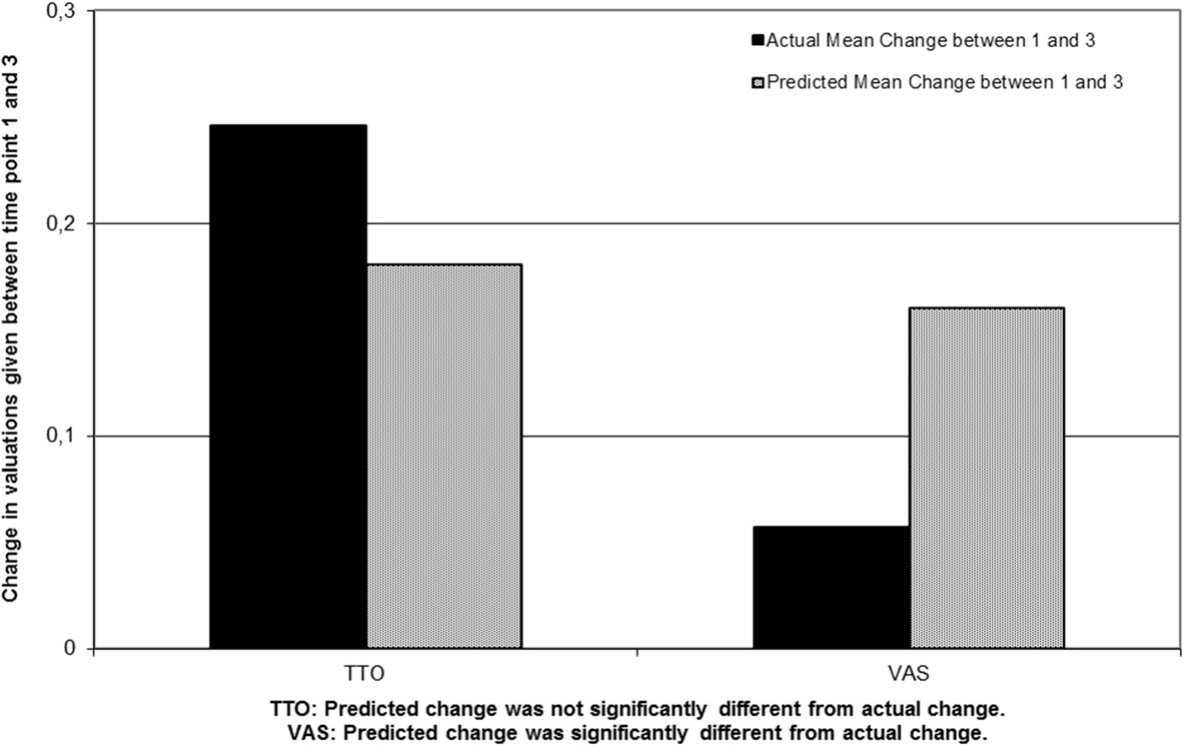
At group level actual change and predicted change at time point 1 and 3 for valuations given for the own health on the Time Trade-Off (TTO) and Visual Analogue Scale (VAS) by patients with Spinal Cord injury.

**Figure 2:**
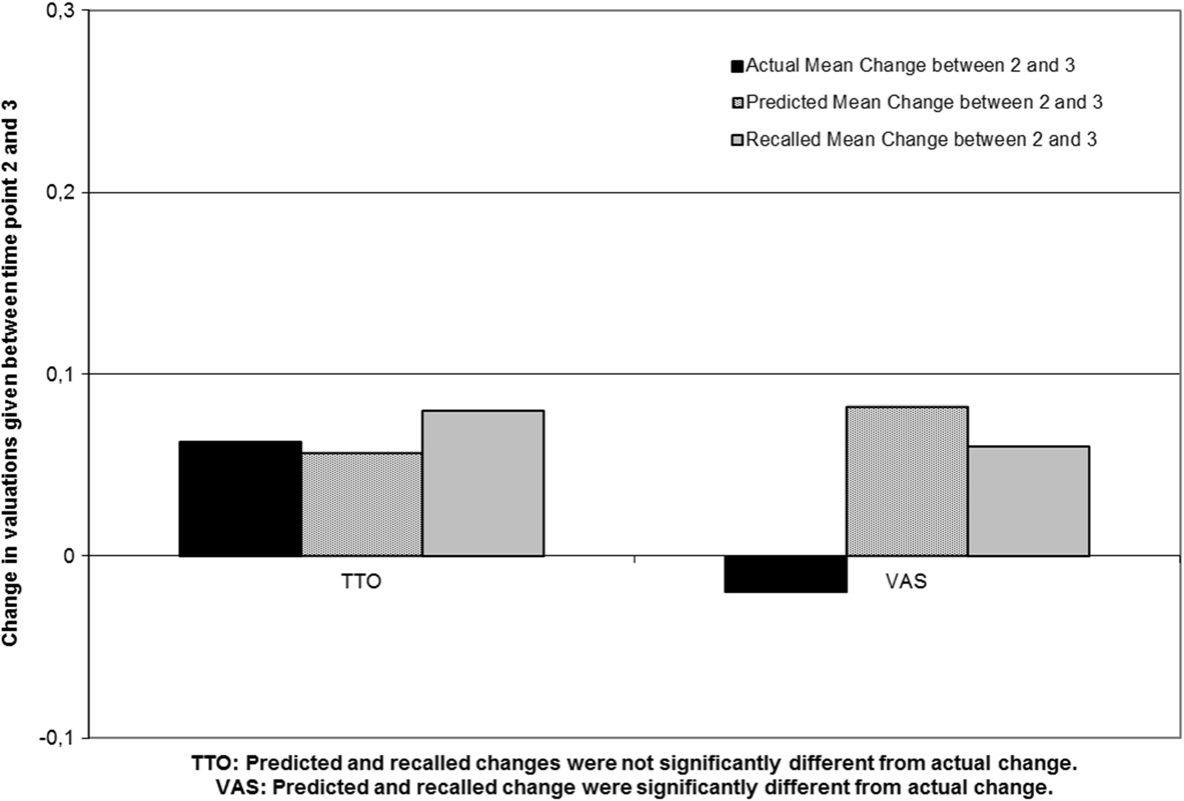
At group level actual change, predicted change and recalled changed between time point 2 and 3 for valuations given for the own health on the Time Trade-Off (TTO) and Visual Analogue Scale (VAS) by patients with Spinal Cord Injury (SCI).

Table 3 shows the mean actual, predicted, and recalled valuations of health states. In Figure 1 and Figure 2 the actual, predicted and recalled mean changes are presented. The VAS shows that at the group level patients over-predicted their future and previous health compared to their actual health. Between the first and third interview patients expected significant improvement (difference d = 0.16 (0.14) (t(43) = -7.39, *p* < 0.001)) but showed only a minor actual improvement (d = 0.06 (0.21) (t(43) = -1.77, *p* = 0.084)). Between the second and third interview, patients again predicted significant improvement (d = 0.08 (0.11) (t(43) = 4.81, *p* < 0.001)) but here their valuations for their actual health even slightly decreased (d = -0.02 (0.19) (t(43) = 0.71, *p* = 0.48)). Finally, they also recalled a small improvement at the group level (d = 0.06 (0.21) (t(43) = 1.91, *p* = 0.06)).

**Table 3:** Accuracy of predicted and recalled valuations of patients with Spinal Cord Injury (SCI) on Time Trade-Off (TTO) and Visual Analogue Scale (VAS) at the group level

	**N**	**T1 actual**	**T2 actual**	**T3 actual**	**T1 *prediction *** **of T3**	**T2 *prediction *** **of T3**	**T3 *recall *** **of T2**	**T1 *prediction *** **of T3 vs T3** *** actual***	**T2 *prediction *** **of T3 vs T3 *acutal***	**T2 *actual *** **vs T3 r** ***ecall *** **of T2**
		**Mean (SD)**	**Mean (SD)**	**Mean (SD)**	**Mean (SD)**	**Mean (SD)**	**Mean (SD)**	***t-value***	***t-value***	***tcxx value***
TTO	44	0.43	0.62	0.69	0.61	0.68	0.61	1.48	0.11	0.44
		(0.33)	(0.31)	(0.29)	(0.31)	(0.30)	(0.30)			
VAS	44	0.60	0.67	0.65	0.76	0.75	0.59	3.24*	3.44**	3.26*
		(0.18)	(0.15)	(0.20)	(0.13)	(0.14)	(0.18)			

Ratings of patients with SCI for their actual and predicted state of health on a TTO and a VAS, at baseline, a few weeks before discharge and six months after discharge. Actual change is compared with expected change using student’s t-test.

**p < .001, *p = < .01.

For the TTO on the other hand, at a group level patients gave accurate predictions and had accurate recollections when compared to the actual change. Between the first and third interview patients predicted an improvement of d = 0.18(0.22) (t(43) = 5.36, *p* <0.001) and their state of health actually improved by d = 0.25 (0.30) (t(43) = -5.43, p < 0.001). Between the second and third interview patients predicted an improvement of d = 0.06 (0.09) (t(43) = 4.27, p < 0.001) and their state of health actually showed an improvement of d = 0.06 (0.30) (t(43) = 1.40, *p* = 0.17)) at the group level. Finally, they also recalled a small improvement at the group level (d = 0.08 (0.22)(t(43) = 2.33, *p* = 0.025)). Both the predicted and the recalled valuations did not differ significantly from the actual change (Table 3).

At the individual level 41% of the respondents gave an accurate prediction of the VAS at first interview for their health at the third interview. At the second interview the correct prediction was slightly lower, 37%. Finally, 41% recalled their health during the period of the second interview correctly. For the TTO the percentages were similar, at the first interview 43% gave accurate predictions, at the second interview this was 39% and at the third interview 45% recalled their health during the period of the second interview correctly. Comparing the valuations given on the VAS with valuations given on the TTO it seems that respondents on the VAS more often over predicted and under recalled their improvement (Table 4). This is in line with the findings at the group level.

**Table 4:** Accuracy of predicted and recalled valuations of patients with Spinal Cord Injury (SCI) on Time Trade-Off (TTO) and Visual Analogue Scale (VAS) at the individual level

	**N**	**T1 *prediction *** **of T3 vs T3 *actual***			**T2 *prediction *** **of T3 vs T3 *acutal***			**T2 *actual *** **vs T3 r** ***ecall *** **of T2**		
		**Under prediction**	**Accurate**	**Over prediction**	**Under prediction**	**Accurate**	**Over prediction**	**Under recall**	**Accurate**	**Over recall**
TTO	44	30%	43%	27%	25%	39%	36%	34%	45%	21%
VAS	44	14%	41%	45%	11%	37%	52%	48%	41%	11%

Under prediction = ∆ prediction - actual < -0.10.

Accurate = ∆ prediction - actual ≤ 0.10 and ≥ - 0.10.

Over prediction = ∆ prediction - actual > 0.10.

Under recall = ∆ recall - actual < -0.10.

Accurate = ∆ recall - actual ≤ 0.10 and ≥ - 0.10.

Over recall = ∆ recall - actual > 0.10.

**Figure 3:**
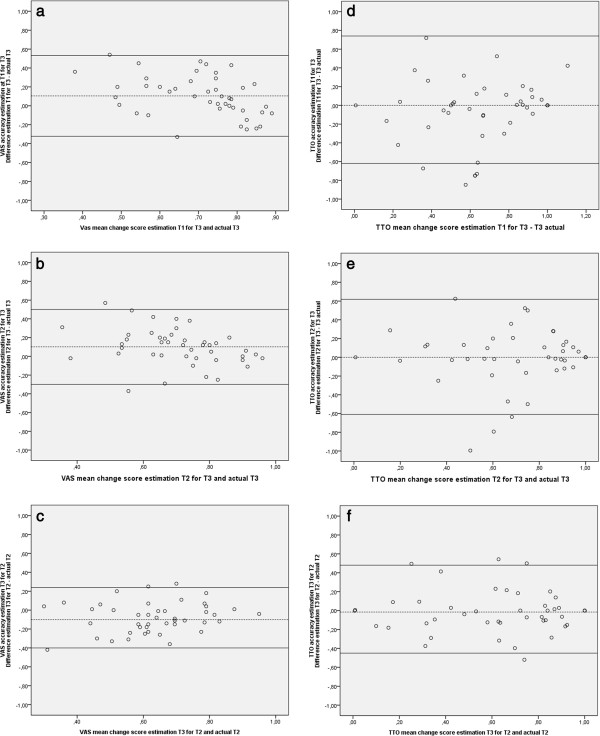
Individual accuracy between predicted health at T1 for actual health at T3, predicted health at T2 for actual health at T3 and recalled health at T3 for actual health at T2 for VAS and TTO.

The Bland-Altman plots (Figure 3) show the 95% confidence interval of the accuracy. The 95% confidence interval for the VAS of T1 prediction of T3 vs T3 actual is -0.32 -0.53, for T2 prediction of T3 vs T3 actual is -0.30-0.50, and for the T2 actual vs T3 recall of T2 is -0,40 -0.24. The 95% confidence interval of the TTO of T1 prediction of T3 vs T3 actual is -0,62 -0,74, for T2 prediction of T3 vs T3 acutal is -0,61 – 0,62, and for the T2 actual vs T3 recall of T2 is -0,45-0,48.

## Discussion

The aim of our study was to attempt to disentangle scale recalibration and ability to anticipate one’s adaptation, in patients with SCI. We explored whether patients correctly predict their future QoL and recall their previous QoL at the group level. In an effort to disentangle scale recalibration and poor anticipated adaptation we used a then-test approach [22] with valuations given on a TTO and VAS. As hypothesized, at the group level the predicted and recalled improvements on the TTO were similar to the actual improvements. On the VAS patients expected an improvement and recalled improvement, but according to their actual ratings they remained stable. Scale recalibrations were likely to have occurred on the VAS. Over time, patients thus used different standards on the VAS. This is in line with the study of Lacey et al [7] who found proof for the effect of scale recalibration on valuations on the VAS. In contrast, other research, reported that similar differences on the VAS should be explained by recall bias [24]. It seems preliminary to conclude that the change on the VAS is fully caused by scale recalibration or by recall bias. When a then-test approach is used, recall bias and response shift are almost impossible to disentangle in the field of QoL [29-31]. In the field of education, the differences between recall bias and response shift can be disentangled since in this field subjective ratings can be compared by objective tests measuring the change in knowledge [32]. Another explanation for our findings is that patients in this study were simply over optimistic about their recovery, possibly based on their theories of stability and change [33]. However, if patients were overly optimistic about their recovery, or if the findings were led by recall bias, we would have expected to find similar results on the TTO.

Regarding to the TTO, patients at the onset of a disability were able to predict their own ability to adapt; they accurately predicted their valuations on the TTO for their future health states and recalled their previous health. Patients were able to estimate correctly their increase in QoL in the first part of their rehabilitation trajectory and the lack of increase in the second part of their rehabilitation trajectory. Previously it was found that during rehabilitation patients with SCI do adapt to their situation both physically as psychologically [34]. In this study we find that at the group level patients accurately estimate the effect of physical and psychological adaptation on their future QoL, where the concept 'adaptation’ refers to aspects such as learning how to handle a wheelchair, finding new hobbies and learn how to live with the new situation, reflecting reprioritization and reconceptualization in terms of quality of life measurement. Compared to a population without any experience of a disability [35], only even limited experience with a disability seems to be sufficient for accurate predictions on a TTO. Even shortly after injury, patients adapt to their disability [34,36]. The insight into their ability to adapt may have enabled them to anticipate accurately. In contrast, people without any experience of a disability find it difficult to incorporate their ability to adapt [35] even after an exercise making them aware of this adaptation [37]. Obviously, these conclusions are restricted to our findings at the group level.

Utility assessment is only used at the group level, generally for cost-effectiveness analyses, but also for calculation of quality-adjusted life years for guideline development. The methods have insufficient reliability for individual level decision making [21]. Therefore, our main interest lies with the paired comparisons. Most response shift research is based on findings at a group level [38-41] although some researchers have examined individual changes [42,43] The findings at the individual level show that only between 37% and 45% of the respondents were accurate in their predictions. These findings are in line with previous research, McPhail and Haines found that 40% of their respondents made correct predictions [38]. In contrast to the group level data, at the individual patient level the accuracy of the predictions was similar for the TTO (39-45%) and the VAS (37-41%) when we made a restriction up to a difference of 0.100. However, the Bland-Altman plots show that the deviation of the accuracy is larger for the TTO then the VAS. That is, the accuracy of 95% of the respondents was lower in the TTO then in the VAS. The distributions of the errors were uneven for the VAS, though, leading to a difference at the group level. On the VAS, predictions of future health were more often higher than the original valuations and predictions of recalled health were more often lower than the original valuations. This is also in line with what can be expected when ratings are influenced by scale recalibration. After a stressful experience people tend to change their internal standards [6]. If patients with SCI have changed their standards at post-test their valuation of their QoL on the then-test will be lower than the original valuation at pre-test [41]. So despite the poor accuracy at the individual level, the differences in results between the TTO and the VAS seem to point to scale recalibration. We urge others, however, to perform more research on predictions and recall using TTO and VAS, for a proper interpretation of these findings.

The results at the group level in this study are retrieved by using the then-test and the results at the individual level were based on Bland-Altman plots and percentages accuracy. These methods are limited, besides the effect of bias [31] such as recall bias [22] a then test is also influenced by an order effect [44]. To further investigate anticipated adaptation and the effect of scale recalibration on the TTO and the VAS statistical methods should be used in which group and individual effects are analyzed simultaneously [8,45]. Herefore, the guidelines to investigate response shift as described by Schwartz et al. [8] could be helpful.

Without objective valuations or a so-called 'gold standard’, our findings cannot implicate that valuations given on the TTO are more accurate than valuations given on the VAS. The only implication that may follow from our findings is, that for valuations over time and when subject samples are compared, the VAS seems vulnerable to scale recalibration at the group level [22]. This might lead to less consistent comparisons when using the VAS than when using the TTO. However, the TTO contains other challenges. For instance, people with low numeracy have difficulty answering the TTO [46]. Our data also show that the TTO has more variance between respondents compared to the VAS. This variance directly influences the power of our analyses. The power in both methods is low, due to the rather small sample size of this study. This small sample size is mostly due to practical reasons. First of all, the prevalence of acute SCI is low, but more importantly, we used a longitudinal design. Over time, 16 patients dropped out. It is important to replicate these findings in a larger sample.

In this study, patients with SCI were interviewed shortly after their injury took place. During this period patients are going through an intensive rehabilitation process. Given the rehabilitation process, patients were interviewed in a rehabilitation center. Naturally, this caused some limitation to their ratings. Although patients reported they were able to assess their physical, social and psychological functioning in this unusual situation, the data cannot be generalized to other patient samples. Besides the setting, the order of health state valuations might have influenced our findings as well [47]. Patients first rated their current health, followed by a valuation of their future health (or in the last interview of their previous health). Their current health might have had influence on how the patients have rated their future or previous health. Given that patients improved over time, their ratings of their actual health at post-test will have been higher compared to their then-ratings, which might have negatively influenced the latter. Investigating aspects of response shift among patients with a disability might seem questionable. One might assume that patients with a disability find a certain balance after going through rehabilitation [48]. However, we examined the aspects of response shift in the subinitial phase after injury. From a related study we know that these patients do go through physical as well as psychological adaptation [34]. Moreover it is suggested that response shift is an important aspect of rehabilitation [49]. In his paper van Rijn [49] explains the effect of response shift on the way people adequately adapt to their spinal cord injury.

In this study we assumed that differences in results between TTO and VAS could be attributed to scale recalibration in the VAS. However, the assumption that the TTO is less vulnerable to scale recalibration is only theoretical, we do not have empirical evidence. It might be possible that other underlying causes led these findings. Future research should further examine the effect of scale recalibration on the TTO. It might be possible that other underlying causes led these findings.

## Conclusion

We tentatively conclude that the health state valuations given on the TTO reveal that patients with SCI in the subinitial phase of their rehabilitation accurately anticipate their adaptation. They accurately predict the effect of change in values, goals, activities and learning new skills, such as in using a wheelchair, on their QoL. Valuations given on the VAS seem biased by scale recalibration, although we need to remind the reader that the results are preliminary. Theoretically, this paper assumes that the three aspects of response shift can be disentangled, with reconceptualization en reprioritization as aspects of adaptation and scale recalibration as measurement bias. However this point of view has been discussed previously [7,11-14] and needs further empirical and theoretical investigation.

## Abbreviations

TTO: Time trade-off; VAS: Visual analogue scale; QoL: Quality of life; RC: Rehabilitation center; SCI: Spinal cord injury.

## Competing interests

The authors declare that they have no competing interests.

## Authors’ contributions

YEP participated in the design of the study, collected the data, performed the statistical analyses, interpreted the findings and wrote the first draft of the manuscript. AMS participated in the design of the study, interpreted the findings and helped to draft the manuscript. Both authors read and approved the final manuscript.
